# Supplementary material for: Magnetic susceptibility properties of tumor-associated cells imaged by MRI reveal glioblastoma infiltration in the edema region
Source: Commun Med (Lond). 2025 Nov 20;5:487. doi: 10.1038/s43856-025-01177-y (PMC12635280; doi:10.1038/s43856-025-01177-y)
Supplement: Supplementary file 2 — Supplementary Information [file 43856_2025_1177_MOESM2_ESM.pdf]

## Supplementary Information

### Supplementary Methods

*Details about brain tumor patients in-vivo MRI, ex-vivo MRI and histochemical analysis*

A 3T MRI scanner equipped with a 48-channels (uMR890, United Imaging Healthcare, Shanghai, China) was used to acquire the patients' images. For diagnostic and presurgical MRI, besides conventional structural images, a 3D multi-echo GRE sequence with the following parameters was acquired: six echoes $TE_1/\Delta TE=2.5/4.3$ ms, TR=35 ms, FA=15°, voxel size=1.03x1.03x2 $mm^3$, FOV=224x224 $mm^2$, 70 slices. Following a comprehensive clinical assessment, the patients underwent surgical resection. The tumor specimen was immediately fixed in a 4% paraformaldehyde solution after excision. After fixation at 4 °C for four weeks, the specimen was transferred into 0.01 M phosphate-buffered saline (1×PBS) for 48 hours to allow for signal recovery prior to MR scanning. For MRI acquisition, the specimen was placed in a 50-mL centrifuge tube and fully immersed in liquid fluorocarbon (Galden, PFPE; Solvay, Brussels, Belgium). MRI was performed using a 9.4T MRI scanner (Bruker BioSpec 94/30, Ettlingen, Germany) with a 4-channel cryoprobe to enhance the signal-to-noise ratio. The specimen was positioned such that its long axis aligned with the direction of the main magnetic field. High-resolution 3D multi-echo GRE images were acquired with an isotropic spatial resolution of 100 μm. Other imaging parameters include: seven echoes $TE_1/\Delta TE=3.2/4.4$ ms, TR=150 ms, FA=25°. The same tissue specimens were processed for histochemical analysis following MRI acquisition. The tissues were cryoprotected in 15% and then 30% sucrose solutions at 4 °C until they sank, indicating complete cryoprotection. Once fully cryoprotected, the specimen was embedded in optimal cutting temperature compound and stored at −80 °C until ready for sectioning. The specimens were sectioned at a thickness of 40 μm using a cryostat (Leica CM1950, Wetzlar, Germany), with sections cut to maximize the cross-sectional area. Consecutive sections were rinsed with 1×PBS prior to staining. One tissue section containing visible tumor tissue was first subjected to hematoxylin and eosin (H&E) staining. Adjacent sections were used for DAB enhanced Perls' staining, which was carried out for 20 minutes at 37 °C to visualize iron distribution. Whole-section images were captured using an upright microscope (Leica DM6 B, Wetzlar, Germany) at a magnification of 5×.

**Supplementary Figures**

**Supplementary Figure 1: Representative PCS slices of the patients of the present study**

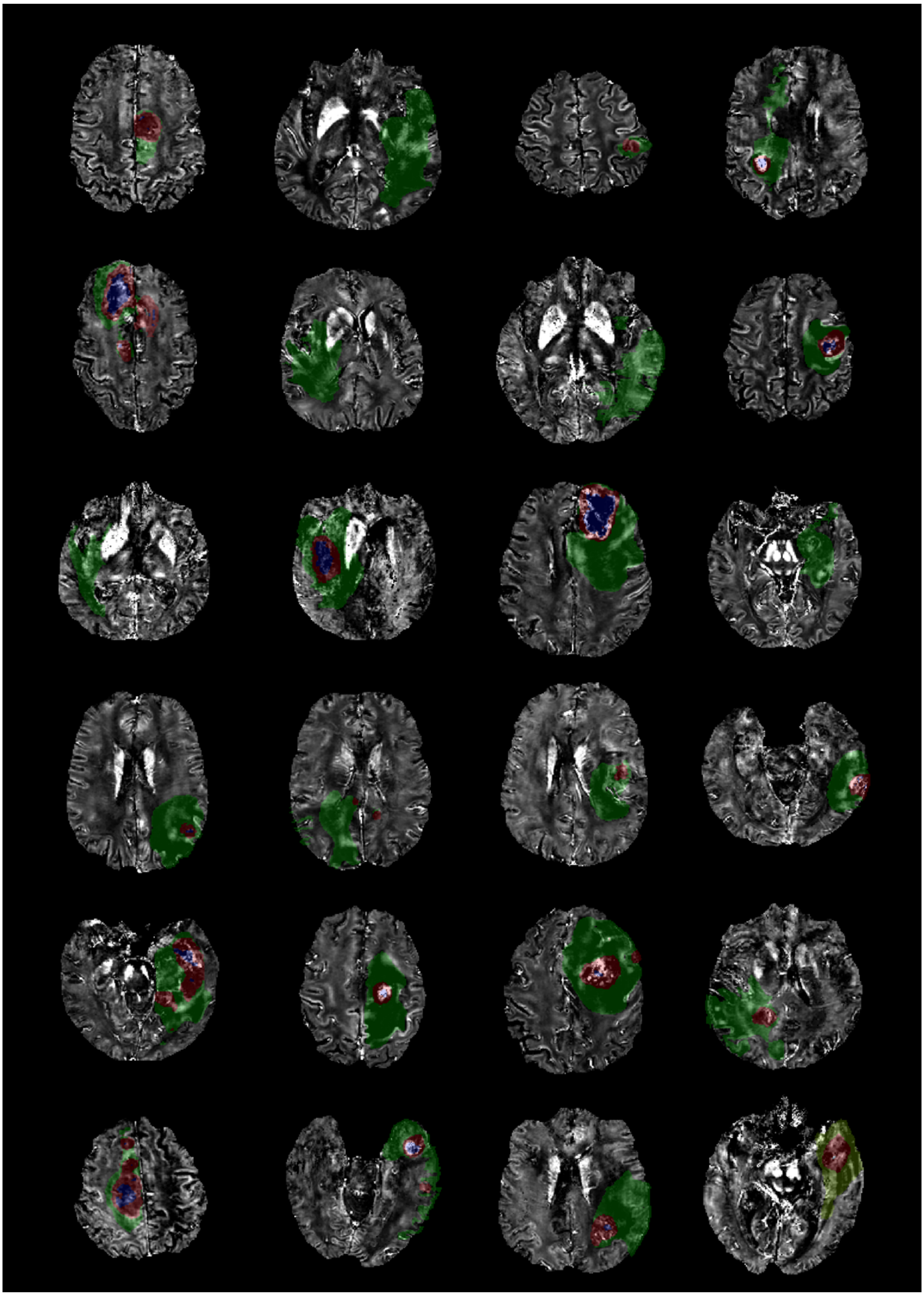

PCS maps are displayed in grayscale colormap which ranges from 0 (black) to 0.07 (white) ppm. The tumor mask is overlapped in color scale (green: edema; blue: necrosis; red: contrast-enhancing tissue).

**Supplementary Figure 2: PCS hyperintensity is not caused by artifacts of QSM reconstruction**

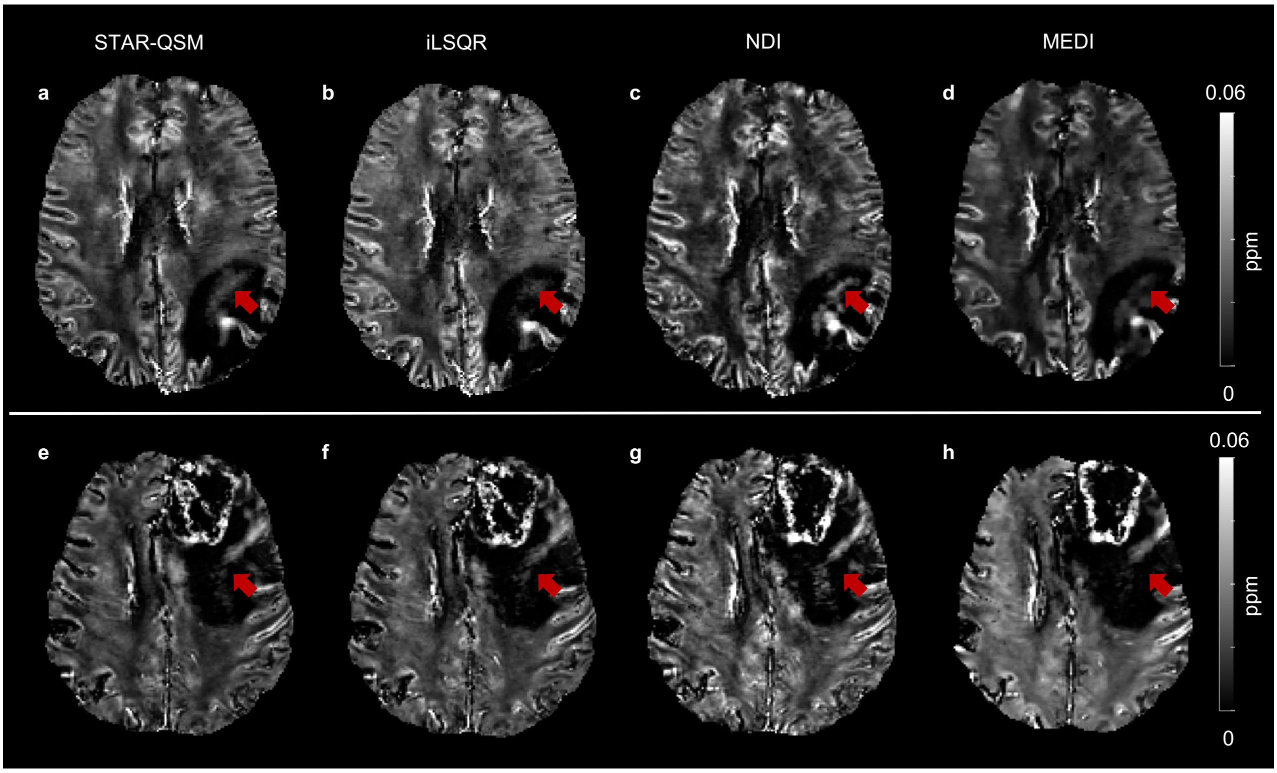


PCS maps of two representative subjects (top and bottom row, respectively) obtained by using STAR-QSM (**a,e**), iLSQR (**b,f**), Non-linear Dipole Inversion (NDI)[1] (**c,g**) and Morphology Enabled Dipole Inversion (MEDI)[2] (**d,h**) algorithms for QSM dipole inversion step. The red arrow points at the PCS hyperintensity. As expected, different QSM algorithms result in slightly different QSM values (e.g., the difference between STAR-QSM and iLSQR derived values is within 2%[3]) thus different PCS values; nonetheless, the PCS hyperintensity remains visible in all cases.

[1]Polak, D. et al. Nonlinear dipole inversion (NDI) enables robust quantitative susceptibility mapping (QSM). NMR Biomed. 33, e4271 (2020).

[2]Liu, T. et al. Morphology enabled dipole inversion (MEDI) from a single angle acquisition: comparison with COSMOS in human brain imaging. Magn. Reson. Med. 66, 777–783 (2011).

[3]Wei, H. et al. Streaking artifact reduction for quantitative susceptibility mapping of sources with large dynamic range. NMR Biomed. 28, 1294–1303 (2015).

**Supplementary Figure 3: PCS hyperintensity is still visible after surgery**

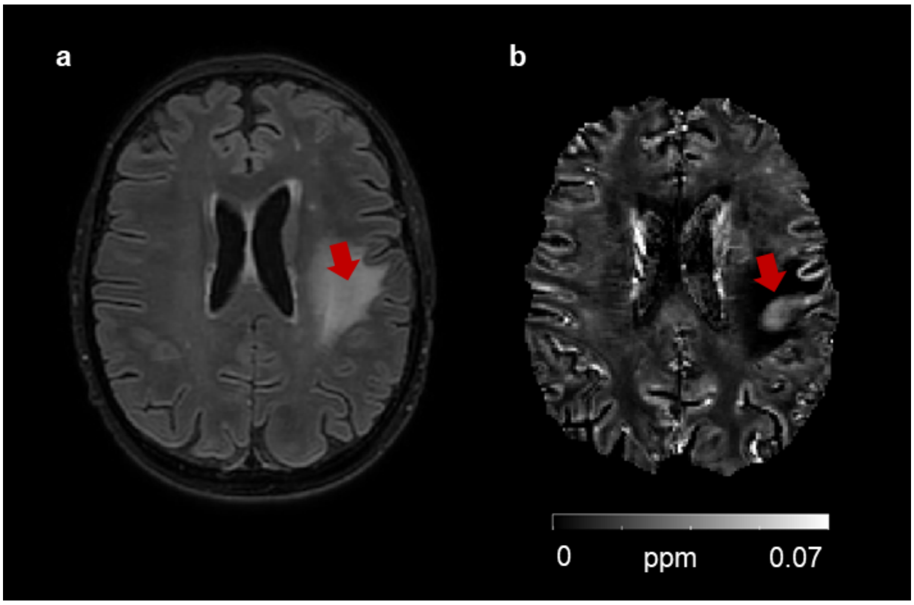


Images of a representative subjects acquired 3 months after surgery. The hyperintensity on the PCS (**b**) and the corresponding area on the FLAIR (**a**) are indicated by red arrows. The PCS map is eroded with respect to the FLAIR image, but it is a well-known consequence of QSM processing.

**Supplementary Figure 4: PCS hyperintensity is not caused by artifacts of the DECOMPOSE algorithm**

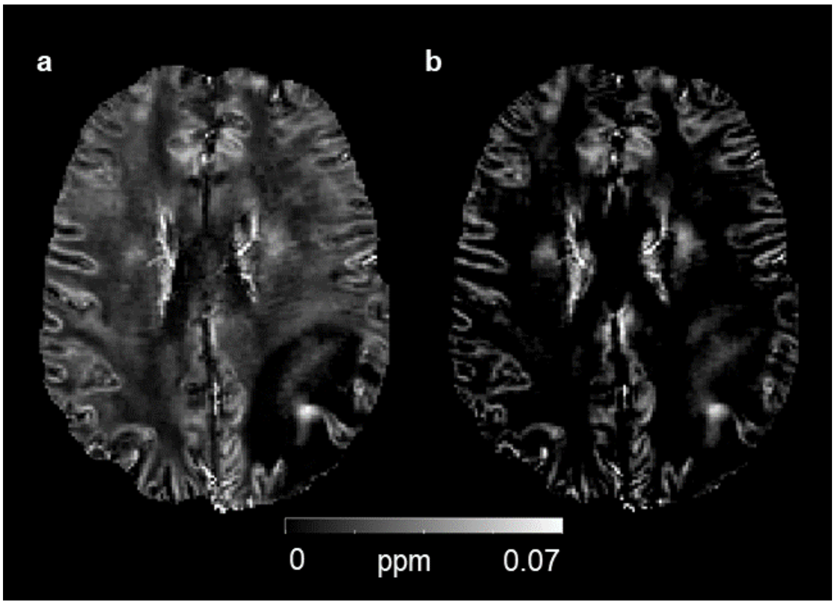


**a**, PCS map and **b**, QSM>0 for a representative subject.

**Supplementary Figure 5: Blood contribution to paramagnetism varies across the tumor regions**

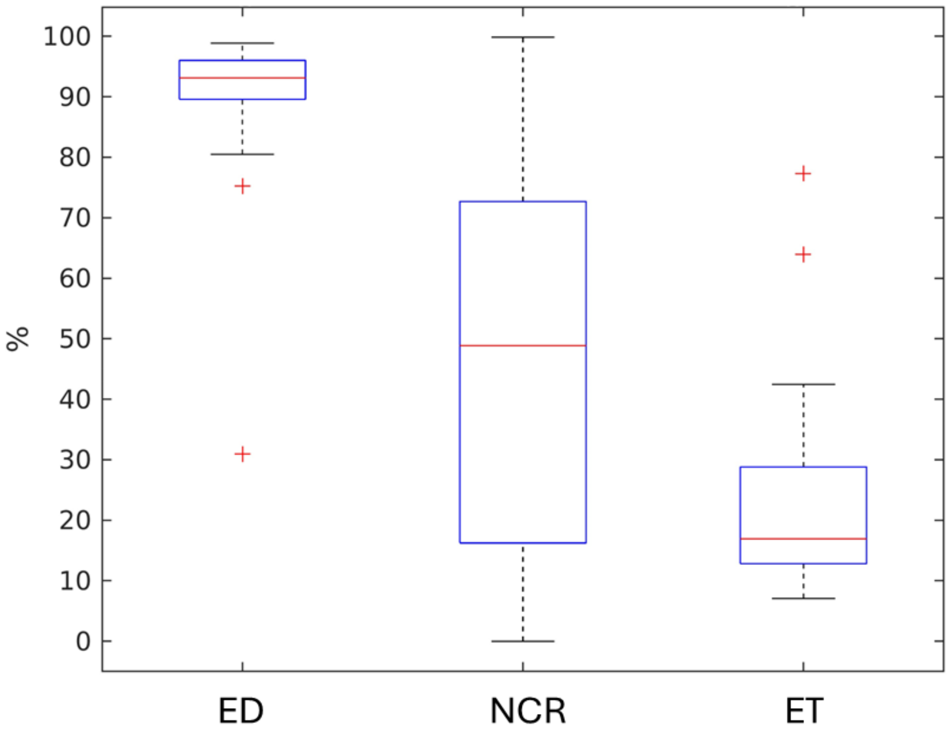


Percentage of voxels belonging to the tumor mask that are not deleted during the process of elimination of voxels dominated by blood. Each boxplot represents one tissue of the tumor, namely edema (ED), necrosis (NCR) and enhancing tissue (ET).
