# Supplementary material for: Magnetic susceptibility properties of tumor-associated cells imaged by MRI reveal glioblastoma infiltration in the edema region
Source: Commun Med (Lond). 2025 Nov 20;5:487. doi: 10.1038/s43856-025-01177-y (PMC12635280; doi:10.1038/s43856-025-01177-y)
Supplement: Supplementary file 3 — Reporting summary [file 43856_2025_1177_MOESM3_ESM.pdf]

nature portfolio

Corresponding author(s): Prof. Chunlei Liu

Last updated by author(s): Sep 12, 2025

# Reporting Summary

Nature Portfolio wishes to improve the reproducibility of the work that we publish. This form provides structure for consistency and transparency in reporting. For further information on Nature Portfolio policies, see our Editorial Policies and the Editorial Policy Checklist.

## Statistics

For all statistical analyses, confirm that the following items are present in the figure legend, table legend, main text, or Methods section.

| n/a | Confirmed | |
|---|---|---|
| ☐ | ☒ | The exact sample size ($n$) for each experimental group/condition, given as a discrete number and unit of measurement |
| ☐ | ☒ | A statement on whether measurements were taken from distinct samples or whether the same sample was measured repeatedly |
| ☐ | ☒ | The statistical test(s) used AND whether they are one- or two-sided<br>*Only common tests should be described solely by name; describe more complex techniques in the Methods section.* |
| ☐ | ☒ | A description of all covariates tested |
| ☐ | ☒ | A description of any assumptions or corrections, such as tests of normality and adjustment for multiple comparisons |
| ☐ | ☒ | A full description of the statistical parameters including central tendency (e.g. means) or other basic estimates (e.g. regression coefficient) AND variation (e.g. standard deviation) or associated estimates of uncertainty (e.g. confidence intervals) |
| ☒ | ☐ | For null hypothesis testing, the test statistic (e.g. $F$, $t$, $r$) with confidence intervals, effect sizes, degrees of freedom and $P$ value noted<br>*Give P values as exact values whenever suitable.* |
| ☒ | ☐ | For Bayesian analysis, information on the choice of priors and Markov chain Monte Carlo settings |
| ☒ | ☐ | For hierarchical and complex designs, identification of the appropriate level for tests and full reporting of outcomes |
| ☒ | ☐ | Estimates of effect sizes (e.g. Cohen's $d$, Pearson's $r$), indicating how they were calculated |

*Our web collection on statistics for biologists contains articles on many of the points above.*

## Software and code

Policy information about availability of computer code

| | |
|---|---|
| Data collection | Structural images pre-processing was performed with FSL (v6.0.3), ANTs (v2.4.3), SPM12 and MASS (v1.1.0).<br>Tumor segmentation was obtained with a publicly available toolbox (https://gitlab.com/picture-production/picture-nnunet-package/-/tree/main).<br>Magnetic susceptibility maps were computed with the publicly available STISuite (v3.0, https://people.eecs.berkeley.edu/?chunlei.liu/software.html), while DECOMPOSE-QSM algorithm is published (v0.6.6, https://doi.org/10.1016/j.neuroimage.2021.118477), but it is available upon request.<br>Perfusion data was quantified with in-house toolbox available at (https://github.com/FAIR-Unipd/dsc-mri-toolbox).<br>Permeability data was quantified with the publicly available ROCKETSHIP toolbox (https://github.com/petmri/ROCKETSHIP).<br>Diffusion data pre-processing was carried out with MRtrix3 (v3.0.4, https://www.mrtrix.org/). |
| Data analysis | Statistical analysis was performed in Matlab R2022b. |

For manuscripts utilizing custom algorithms or software that are central to the research but not yet described in published literature, software must be made available to editors and reviewers. We strongly encourage code deposition in a community repository (e.g. GitHub). See the Nature Portfolio guidelines for submitting code & software for further information.

# Data

Policy information about availability of data

All manuscripts must include a data availability statement. This statement should provide the following information, where applicable:

- Accession codes, unique identifiers, or web links for publicly available datasets
- A description of any restrictions on data availability
- For clinical datasets or third party data, please ensure that the statement adheres to our policy

Data are available upon reasonable request from the corresponding author with an approved data agreement.

# Human research participants

Policy information about studies involving human research participants and Sex and Gender in Research.

| | |
|---|---|
| Reporting on sex and gender | The patient cohort is composed of 18 males and 6 females, as reported in Table 1 of the manuscript. No subgroup analysis based on sex was performed due to the limited sample size. For the independently acquired patients, relevant information are reported in Supplementary Methods. |
| Population characteristics | Relevant population characteristics are reported in Table 1 and include sex, age at diagnosis and MGMT methylation information. |
| Recruitment | Patients were recruited at the University Hospital of Padova (Padova, Italy) and provided informed, written consent in accordance with the approved study protocol (protocol number: AOP2971). For the two independently acquired patients, informed, written consent in accordance with the Ethics Committee of Ruijin Hospital Luwan Branch, Shanghai Jiao Tong University School of Medicine was obtained. |
| Ethics oversight | The study protocol was approved by the local Ethics Committees. |

Note that full information on the approval of the study protocol must also be provided in the manuscript.

# Field-specific reporting

Please select the one below that is the best fit for your research. If you are not sure, read the appropriate sections before making your selection.

☒ Life sciences ☐ Behavioural & social sciences ☐ Ecological, evolutionary & environmental sciences

For a reference copy of the document with all sections, see nature.com/documents/nr-reporting-summary-flat.pdf

# Life sciences study design

All studies must disclose on these points even when the disclosure is negative.

| | |
|---|---|
| Sample size | This is a pilot discovery study for which we did not compute any sample size in advance. Additionally, no prior information exists about the effect size. |
| Data exclusions | No data were excluded for the analysis. |
| Replication | The PCS hyperintensity and the consequent elevated PDR area were consistently observed in all patients. Susceptibility maps were computed with two different dipole inversion algorithms and derived PCS maps were similar, as can be appreciated in Supplementary Figure S2. |
| Randomization | Experimental groups are not needed for the purpose of our study. |
| Blinding | Blinding was not relevant for our study since no experimental groups were present. |

# Reporting for specific materials, systems and methods

We require information from authors about some types of materials, experimental systems and methods used in many studies. Here, indicate whether each material, system or method listed is relevant to your study. If you are not sure if a list item applies to your research, read the appropriate section before selecting a response.

## Materials & experimental systems

| n/a | Involved in the study |
|---|---|
| ☒ | ☐ Antibodies |
| ☒ | ☐ Eukaryotic cell lines |
| ☒ | ☐ Palaeontology and archaeology |
| ☒ | ☐ Animals and other organisms |
| ☐ | ☒ Clinical data |
| ☒ | ☐ Dual use research of concern |

## Methods

| n/a | Involved in the study |
|---|---|
| ☒ | ☐ ChIP-seq |
| ☒ | ☐ Flow cytometry |
| ☐ | ☒ MRI-based neuroimaging |

# Clinical data

Policy information about clinical studies

All manuscripts should comply with the ICMJE guidelines for publication of clinical research and a completed CONSORT checklist must be included with all submissions.

| | |
|---|---|
| Clinical trial registration | This is not a clinical trial, it is a research study that is using clinical data. |
| Study protocol | This is not a clinical trial, it is a research study that is using clinical data (protocol number: AOP2971). |
| Data collection | Data are stored in a controlled access data storage and recruitment took place at the University Hospital of Padova (Padova, Italy). For the two independently acquired patients, data collection took place at the Ruijin Hospital Luwan Branch, Shanghai Jiao Tong University School of Medicine. |
| Outcomes | This is not a clinical trial, it is a research study that is using clinical data. |

# Magnetic resonance imaging

## Experimental design

| | |
|---|---|
| Design type | Structural, susceptibility, perfusion, permeability and diffusion imaging. |
| Design specifications | The same acquisition protocol was used for all patients included in the study. |
| Behavioral performance measures | No behavioral performance measures were collected. |

## Acquisition

| | |
|---|---|
| Imaging type(s) | Structural, susceptibility, perfusion, permeability, diffusion. |
| Field strength | 3T |
| Sequence & imaging parameters | Data were collected on a 3T Philips Ingenia scanner equipped with a 32-channel head-neck coil. For susceptibility quantification, a 3D multi-echo GRE sequence was acquired with the following parameters: eight echoes TE1/ΔTE=5/5 ms, TR=44 ms, FA=25°, voxel size=1x1x1 mm3, FOV=240x240 mm2, 140 slices. The acquisition protocol also comprises conventional structural MRI sequences: (i) 3D T1-weighted (T1w) image pre- and post-gadolinium-based contrast agent injection (T1w-Gd) (TE=3 ms, TR=6.7 ms, FA=8°, voxel size=1x1x1 mm3, FOV=240x240 mm2, 181 slices); (ii) 3D T2-weighted (T2w) image (TE=280 ms, TR=3000 ms, FA=90°, voxel size=1x0.94x0.94 mm3, FOV=256x256 mm2, 181 slices); (iii) 3D Fluid Attenuated Inversion Recovery (FLAIR) image (TE=360 ms, TR=8000 ms, FA=90°, voxel size=1.12x1.12x1.12 mm3, FOV=221x221 mm2 - reconstructed to voxel size=0.56x0.62x0.62 mm3, FOV=400x400 mm2, 326 slices). In addition, multi-shell diffusion MRI protocol for a total of 116 Diffusion Weighted Images (DWIs) (TE=104 ms, TR=3.7 s, FA=90°, voxel size=2x2x2 mm3, FOV=112x122 mm2, 78 slices, multi-band accelerator factor=2) was acquired: 12 images at b=0 s/mm2, 8 gradient directions at b-value=300 s/mm2, 32 gradient directions at b-value=1000 s/mm2 and 64 gradient directions at b-value=2000 s/mm2. Dynamic Susceptibility Contrast (DSC) and Dynamic Contrast Enhanced (DCE) sequences were acquired. DCE sequence involved a variable flip angle technique (VFA) and 80 dynamic scans with the same geometry (TE=1.8 ms, TR=5 s, voxel size=2.4x2.4x5 mm3, FOV=96x96 mm2, 22 slices). Images for T1 mapping using VFA were acquired (FA=5°/10°/15°) and then dynamic DCE images were collected (FA=15°, 80 volumes). DSC acquisition parameters were: TE=40 ms, TR=1.5 s, FA=75°, voxel size=2.4x2.4x5 mm3, FOV=94x94 mm2, 22 slices, 120 volumes.<br>For the two independently acquired patients, in-vivo acquisition details are: a 3T MRI scanner equipped with a 48-channels (uMR890, United Imaging Healthcare, Shanghai, China) was used. Besides conventional structural images, a 3D multi-echo GRE sequence with the following parameters was acquired: six echoes TE1/ΔTE=2.5/4.3 ms, TR=35 ms, FA=15°, voxel size=1.03x1.03x2 mm3, FOV=224x224 mm2, 70 slices. Additionally, for the ex-vivo acquisition, scans were performed using a 9.4T MRI scanner (Bruker BioSpec 94/30, Ettlingen, Germany) with a 4-channel cryoprobe. High-resolution 3D multi-echo GRE images were acquired with an isotropic spatial resolution of 100μm. Other imaging parameters include: seven echoes TE1/ΔTE=3.2/4.4 ms, TR=150 ms, FA=25°. |
| Area of acquisition | Whole brain. |

Diffusion MRI ☒ Used ☐ Not used

Parameters: Multi-shell protocol with 116 DWIs: 12 images at b=0 s/mm2, 8 gradient directions at b-value=300 s/mm2, 32 gradient directions at b-value=1000 s/mm2 and 64 gradient directions at b-value=2000 s/mm2.

## Preprocessing

| | |
|---|---|
| Preprocessing software | Toolboxes used for pre-processing were FSL (v6.0.3), ANTs (v2.4.3) and MRtrix3 (v3.0.4, https://www.mrtrix.org/). Tissue segmentation was obtained with SPM12 and brain extraction with MASS (v1.1.0). |
| Normalization | Data were not normalized since our image analysis is at single-patient level. |
| Normalization template | Data were not normalized to a template. |
| Noise and artifact removal | n/a |
| Volume censoring | n/a |

## Statistical modeling & inference

| | |
|---|---|
| Model type and settings | Wilcoxon rank sum test was used to assess differences between groups of variables, where Pearson's correlation was computed to investigate associations between continuous variables. |
| Effect(s) tested | n/a |

Specify type of analysis: ☐ Whole brain ☒ ROI-based ☐ Both

Anatomical location(s): TIZ defined with the PDR in the lesion area.

| | |
|---|---|
| Statistic type for inference (See Eklund et al. 2016) | Cluster-wise. |
| Correction | No correction for multiple comparisons was applied. |

## Models & analysis

| n/a | Involved in the study |
|---|---|
| ☒ | ☐ Functional and/or effective connectivity |
| ☒ | ☐ Graph analysis |
| ☒ | ☐ Multivariate modeling or predictive analysis |
